# A Case Report on the Rehabilitation of Severely Worn Teeth Using a Custom-Made Cast Post

**DOI:** 10.7759/cureus.30528

**Published:** 2022-10-20

**Authors:** Utkarsh Umre, Shweta Sedani, Pradnya P Nikhade, Akansha Bansod

**Affiliations:** 1 Conservative Dentistry and Endodontics, Sharad Pawar Dental College and Hospital, Datta Meghe Institute of Medical Sciences, Wardha, IND; 2 Dentistry, Sharad Pawar Dental College and Hospital, Datta Meghe Institute of Medical Sciences, Wardha, IND; 3 Prosthodontics, Sharad Pawar Dental College and Hospital, Datta Meghe Institute of Medical Sciences, Wardha, IND

**Keywords:** esthetic restoration, full veneer crowns, cast metal post and core, post and core, prosthetic rehabilitation

## Abstract

Posts have been suggested to strengthen weak endodontically treated teeth against intra-oral forces by transmitting torquing forces within the radicular dentin to supportive tissue along their roots. The case studies that follow show how an interdisciplinary approach was used to use complete veneer crowns after custom cast posts to repair severely damaged treated teeth and restore their appearance and functionality. Coordinated prosthetic and endodontic treatments with careful consideration of the patient's expectations and requirements were crucial for a positive result and patient satisfaction. For a very long period, a cast metal post and core were used to provide the foundation restoration for a prosthetic crown. The cast post and core system has the benefit that the core is a natural extension of the post. It is intended for the post to keep the core restoration, which rebuilds the destroyed coronal structure. In this article, cast post and core and porcelain fused to metal restorations for injured maxillary central incisors are discussed.

## Introduction

When the remaining coronal tooth structure is insufficient to give retention and resistance form for the restoration, the reconstruction of teeth that have undergone endodontic treatment is frequently necessary before the final restoration can be completed [1]. Due to fractures, dental cavities and teeth that have already undergone restoration and endodontic procedures, the majority of root canal-treated teeth have been proved difficult to restore.

There are numerous post-rehabilitation methods and resources accessible today for cases on very little remaining coronal tooth structure. It can be difficult to choose the best post and core system, hence numerous methods and materials are employed for this purpose in clinical practice [2]. Recent years have seen a rise in the importance of esthetic performance of systems, as well as simplicity of application of various post and core systems. However, a system's durability and dependability are always crucial [3].

A variety of post methods and materials are now available to retain final restorations in situ and restore root canal-treated teeth with moderate to severe loss of coronal tooth structure [4,5]. A supplementary post is inserted into the root of a tooth with insufficient structural support when additional retention is necessary to keep the core and coronal restoration in place [6]. In order for the crown to be properly held and resistant and to restore the tooth's function and appearance, the main goal of the post and core is to restore enough coronal tooth structure [7]. The most common type of post used to hold the core in place is metal. Unlike other ready-made systems, cast posts and cores do not need an extra retention mechanism, like pins, to keep the core in place [8]. A post-core restoration is advised when a tooth has undergone severe crown loss, is vulnerable to cervical area fracture, is noticeably discolored, has lost two proximal surfaces, is shorter, has a poor retention form, and is in good periodontal and periapical health [9].

The geometry of the canal and the quantity of remaining tooth structure are two factors that determine whether to use a cast post or a prefabricated post [10]. If a canal needs extensive preparation, it has been claimed that a well-adapted cast post and core will be more retentive than a prefabricated post that does not match the canal pattern [11,12]. The cast post and core, which are designed to withstand torsional loads, are inserted into the prepared root canal space. The following case report is a rehabilitation of severely worn anterior teeth with the help of custom-made cast post and porcelain fused to metal crowns in anterior esthetic zone.

## Case presentation

**Figure 1:**
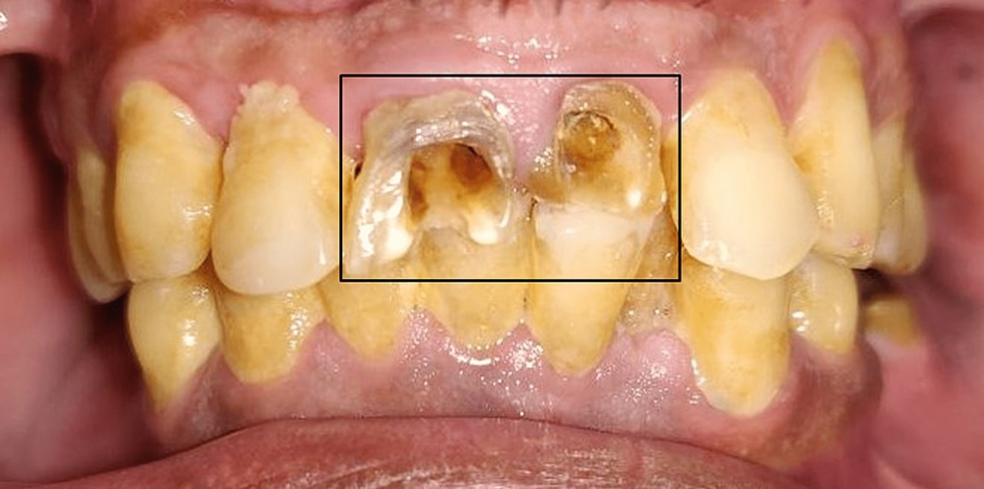
Clinical pre-operative photograph A clinical pre-operative photograph revealed root canal-treated teeth with #11 and #21; the coronal portion was mutilated for both the teeth.

A 42-year-old male patient reported to the Department of Endodontics, Sharad Pawar Dental College and Hospital, Datta Meghe Institute of Medical Sciences, Wardha, India, with the chief complaint of poor esthetics due to dislodged restoration in the upper front region of the jaw and a desire to get them filled. The patient was aware of the gradual esthetic deterioration of his anterior teeth #11 and #21 over many years. Clinical examination revealed secondary caries with dislodged restorations with #11 and #21 as shown in Figure 1.

On radiographic examination, the anterior teeth were endodontically treated. There were no signs of periapical infection and periodontal widening; hence, our goal was to restore the form and function of the lost tooth structure.The treatment plan for the involved teeth included a custom-made cast post and core followed by full coverage porcelain fused metal crowns with #11 and #21.

Clinical steps

**Figure 2:**
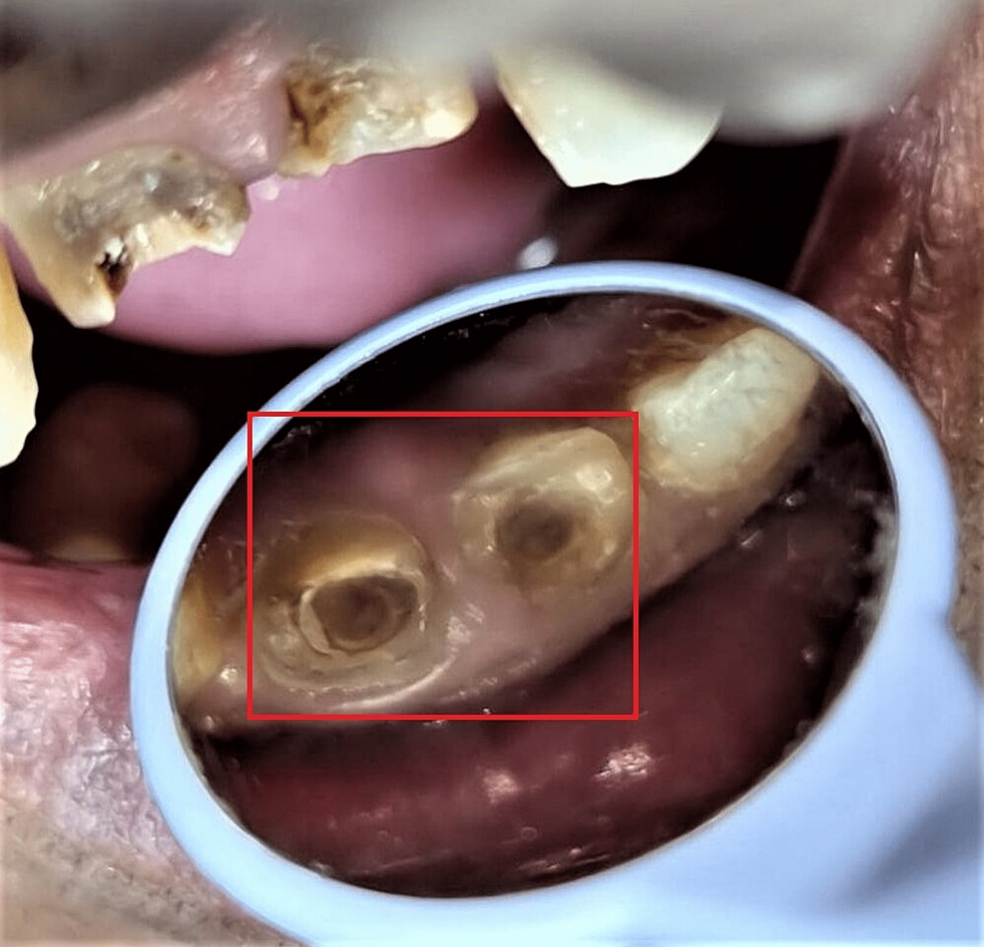
Space preparation for custom-made cast post A minimum of 4 mm of gutta-percha has to be left in the canal for apical seal and preparation was done till #5 peeso reamer.

After removing secondary caries, post space was prepared with 11 and 21 using a peezo-reamer #1 through #4 (MANI Inc., Japan) and an endodontic hand instrument to accept the post. The canal was prepared in a manner to ensure 4 mm of gutta-percha to maintain the periapical seal. The apical seal and post-space preparation were evaluated (Figure 2).

**Figure 3:**
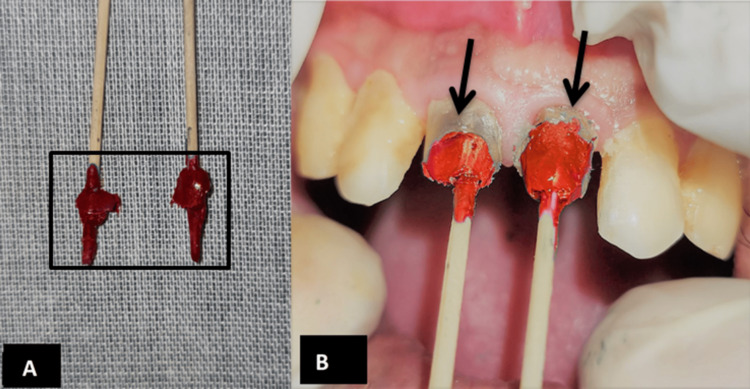
Post-space preparation using pattern resin A: Impressions made with the help of tooth pick using indirect technique; B: Fit of the impressions evaluated intra-orally.

An indirect technique was used for the fabrication of metallic posts. The separating media was applied to the prepared post space, and an impression of 11 and 21 was made with pattern resin (GC America, Pattern resin ) with the help of a toothpick (Figure 3).

**Figure 4:**
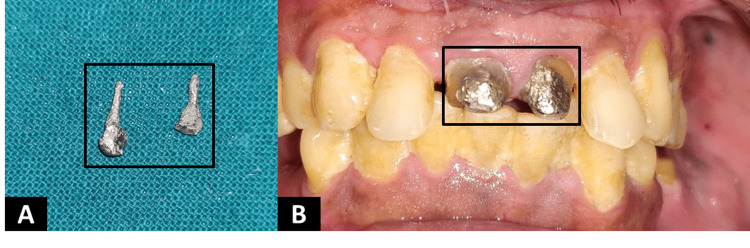
Custom-made cast post fabricated and cemented intra-orally. A: Custom-made cast post fabricated with cobalt-chromium alloy; B: Ferrule preparation on the remaining coronal tooth structure and cemented cast post.

The fabricated post was cemented using zinc-phosphate cement (Prevest Denpro, USA). Following it, the teeth were prepared with a circumferential chamfer, including 1.5 mm of ferrule preparation (Figure 4).

**Figure 5:**
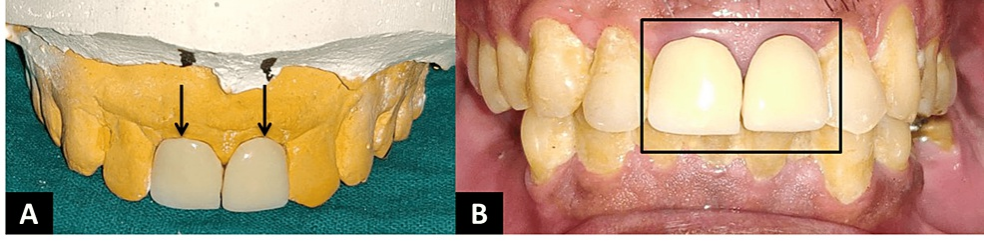
Metal-ceramic crowns with #11 and #21 A: Metal-ceramic crowns seated on the cast; B: Crowns cemented intra-orally with luting glass ionomer cement.

Impressions were made in addition to silicone impression material. Shade selection was done. Porcelain-fused metal crowns were cemented using luting glass ionomer cement (Figure 5).

**Figure 6:**
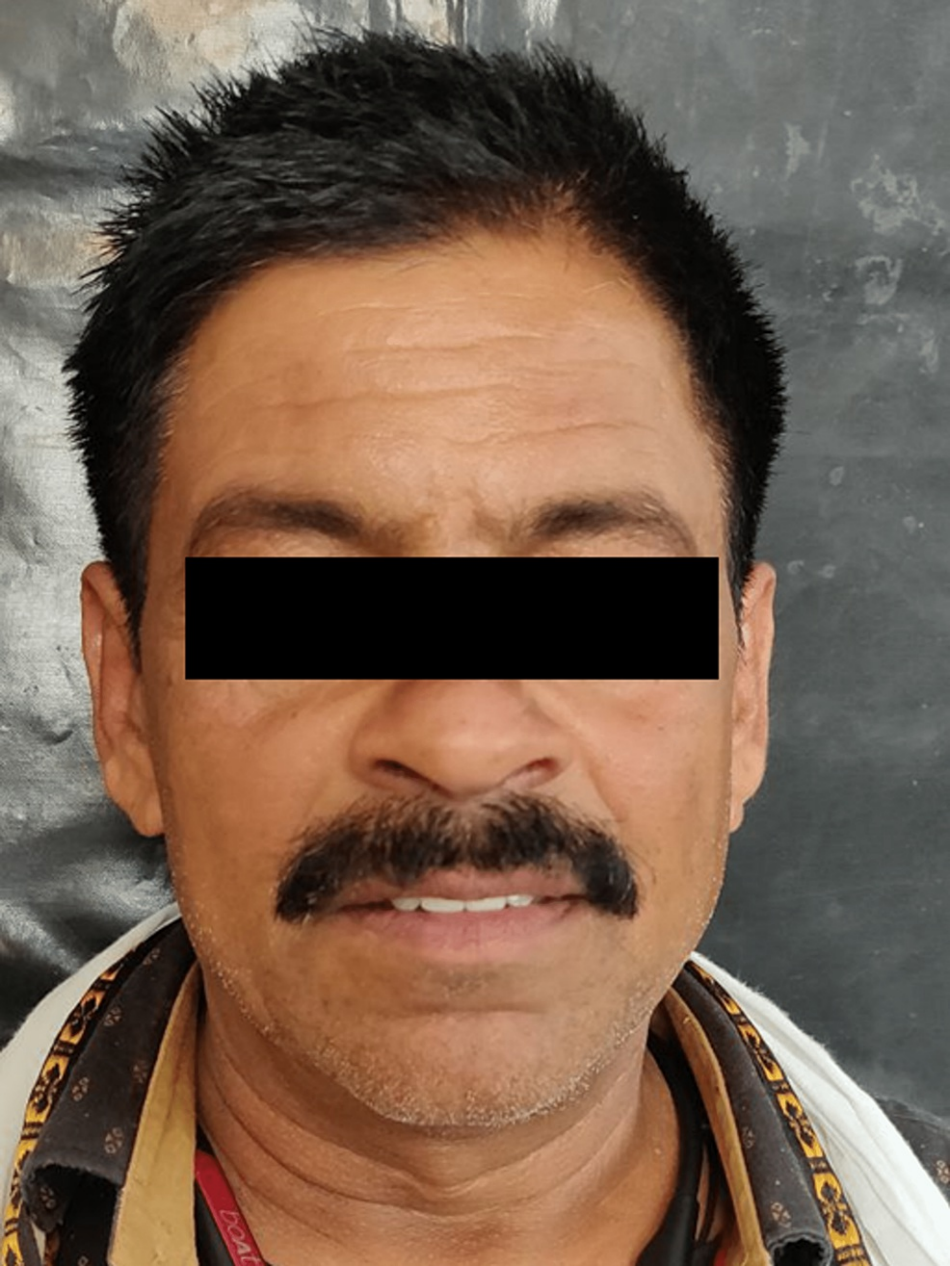
Post-operative extra-oral photograph The patient was satisfied with the esthetic restorations.

The treatment outcome fulfilled the patient's expectations (Figure 6).

## Discussion

For rehabilitation of esthetic and functional teeth, custom cast post fabrication is considered for best retention and resistance. For mutilated teeth, post and core restoration is regarded as the cornerstone procedure. In 2005, Cruegers et al. examined the success rates of cast and metal posts over a period of 60 months and assessed the long-term success rate of metal posts in the follow-ups [11]. In a 109-month follow-up study conducted in 2003, Ellner et al. compared the success rates of cast and metal dowels and discovered that they were comparable in both cases [8]. This procedure's primary goal is maintaining the core restoration, which replaces missing coronal structure, by offering retention. Prefabricated composite or one-piece customized posts and cores both are alternatives [5]. In order to simplify the eventual restoration of the tooth using an indirect extra coronal restoration, the majority of the coronal part of a severely damaged tooth is restored with a post-and-core restoration [13].

For restoring teeth that had previously received endodontic treatment, custom cast posts and cores were thought to be state of the art in this case report. Nowadays, prefabricated posts are frequently chosen over custom cast posts and cores. Cast posts and cores, on the other hand, have their own advantages, such as better flexibility to the root canal, and preservation of the greatest amount of tooth structure. Since the post's core is an intrinsic part, it is not necessary for the post to keep it. An additional feature is an anti-rotational property. However, it has the downside of requiring several visits [14]. Using custom cast posts for root canal and crown preparation advantages include greater strength and minimum tooth structure loss. The strength of a tooth directly relates to the amount of residual tooth structure. Therefore, maximum tooth structure preservation is crucial for the success of post and core restoration [13-15]. On the other hand, prefabricated cylindrical posts chiefly rely on cement for retention. Reduced core retention of the post and the probability of rotation are the major disadvantages of this type of post [3].

There are metal posts (stainless steel, titanium), fibre posts (carbon, glass), and ceramic posts available for the prefabricated posts (Zirconia). The surface characteristics of the prefabricated posts may vary, and they may have a circular cross-section (serrated, smooth, threaded, and roughened). There are parallel and tapered post shapes that can be used, and corresponding drills are included to make post gaps [15].

## Conclusions

The amount and quality of preserved tooth structure, esthetic requirements, as well as their indications, advantages, and disadvantages, should all be taken into consideration when choosing the best post and core systems. There is a vast collection of research that compares the effectiveness and application of various post kinds and the various materials that are used to make them. Although more such studies are required to support the method described in the case report, it is simple, effective, and provides a potential alternative for saving severely damaged or decaying teeth. This process of creating a unique post and core has produced effective outcomes.
